# Identification of the Avulsion-Injured Spinal Motoneurons

**DOI:** 10.1007/s12031-015-0588-4

**Published:** 2015-05-30

**Authors:** Min Tan, Ming-zhou Yuan, Tian-yu Sun, Ying-yu Xie, Lin-Lin Liu, Ying Tang, Ze-min Ling, Ying-qin Li, Guang-yin Yu, Li Hua Zhou

**Affiliations:** Department of Anatomy, Zhongshan School of Medicine, Sun Yat-sen University, No. 74 Zhongshan Road 2, Guangzhou, 510080 People’s Republic of China; Zhongshan School of Medicine, Sun Yat-sen University, No. 74 Zhongshan Road 2, Guangzhou, 510080 People’s Republic of China

**Keywords:** NeuN, ATF-3, Spinal motoneurons, Avulsion, Brachial plexus

## Abstract

In laboratory studies, counting the spinal motoneurons that survived axonal injury is a major method to estimate the severity and regenerative capacity of the injured motoneurons after the axonal injury and rehabilitation surgery. However, the typical motoneuron marker, the choline acetyltransferase (ChAT), could not be detected in the injured motoneurons within the first 3–4 weeks postinjury. It is necessary to explore the useful and reliable specific phenotypic markers to assess the fate of injured motoneurons in axonal injury. Here, we used the fluorogold to retrograde trace the injured motoneurons in the spinal cord and studied the expression patterns of the alpha-motoneuron marker, the neuronal nuclei DNA-binding protein (NeuN) and the peripheral nerve injury marker, the activating transcriptional factor (ATF-3), and the oxidative stress marker, the neuronal nitric oxide synthase (nNOS) within the first 4 weeks of the root avulsion of the right brachial plexus (BPRA) in the adult male Sprague-Dawley rats. Our results showed that ATF-3 was rapidly induced and sustained to express only in the nuclei of the fluorogold-labeled injured motoneurons but none in the unaffected motoneurons from the 24 h of the injury; meanwhile, the NeuN almost disappeared in the avulsion-affected motoneurons within the first 4 weeks. The nNOS was not detected in the motoneurons until the second week of the injury. On the basis of the present data, we suggest that ATF-3 labels avulsion-injured motoneurons while NeuN and nNOS are poor markers within the first 4 weeks of BPRA.

## Introduction

In clinic, the brachial plexus root avulsion (BPRA) is the most serious axonal injury which causes permanent paralysis of the ipsilateral limb muscles. In the studies of the animal model of BPRA, previous studies have proven that the unilateral avulsion of both ventral and dorsal roots of the brachial plexus only affects the ipsilateral ventral horn and caused the death of a majority of the motoneurons which innervate the skeletal muscles of the limbs (Wu 2000; Zhou et al. 2008). Since the motoneurons innervating upper limb muscles reside in the lateral motor column (LMC) at the cervical spinal levels of the mammalian spinal cord (Dasen et al. 2005; Goetz et al. 2015), the number of the motoneurons stained by the traditional neutral red in the LMC of the C5-T1 spinal segments was commonly used to assess the survival and regenerative capacity of the affected motoneurons in BPRA (Oppenheim et al. 1995).

However, recent studies argued the underestimation of the surviving motoneurons by neutral red staining since the severe atrophy but survived neurons would be mistaken as the glia cells (Kwon et al. 2002b; Kwon et al. 2002a). The best way is to count the spinal motoneurons which are stained by the typical motoneuron marker, the choline acetyltransferase (ChAT). Unfortunately, many previous studies have proven that axonal injury results in the absences of ChAT expression in the affected motoneurons within the first 3–4 weeks postinjury (Rende et al. 1995; Hoang et al. 2003; Zhou et al. 2008; Eggers et al. 2010; Peddie and Keast 2011).

Nitric oxide synthase (NOS) is able to generate nitric oxide (NO) by utilizing l-arginine as a substrate and nicotinamide adenine dinucleotide phosphate (NADPH) as the hydrogen delivery body, and the NOS is subdivided into neuronal nitric oxide synthase (nNOS), endothelial nitric oxide synthase (eNOS), and inducible nitric oxide synthase (iNOS) (Forstermann and Sessa 2012). The majority of nNOS is located in the neurons (Wu et al. 1994). Following root avulsion, the nNOS have proven to be induced in the adult rat motoneurons (Wu 1993; Yu 2002). However, the expression of nNOS could only be detected after the first week and then reaches its peak at 3–4 weeks following brachial root avulsion (Wu 1993, 1996; Zhou et al. 2008). Although the expression of the nNOS has been proven to be inside the injured motoneurons and thought to trigger the motoneuron death (Wu 1993; Novikov et al. 1995; Brown 2010), the nNOS is not a good marker for the injured motoneurons since it disappears within the first week and does not appear in all of the injured motoneurons within the first 4 weeks postinjury.

The recent study showed that the panneuronal marker (Mullen et al. 1992), neuronal nuclei DNA-binding protein (NeuN), could mark the alpha motoneurons of the spinal cord of the mice (Friese et al. 2009). We do not know whether NeuN could mark the affected motoneurons of rats or not. Recently, activating transcriptional factor (ATF-3) was considered as the specific regenerative marker of the affected motoneurons since it was found to be persistent in affected and regenerating lumbar motoneurons in axonal injury (Linda et al. 2011), and most importantly, ATF-3 was not induced transsynaptically in spinal dorsal horn neurons (Tsujino et al. 2000). In the study of the BPRA, the cervical motoneurons underwent degeneration if there is no surgical repairing (Oppenheim et al. 1995; Gu et al. 2004; Fu et al. 2014). We ask the question whether ATF-3 could mark the degenerative cervical motoneurons or not. In order to explore a stable and reliable molecular marker of the survival and degenerating spinal motoneurons in BPRA, we investigated and compared the expression patterns of the ATF-3 and NeuN in the cervical motoneurons within 4 weeks of the injury.

## Material and Methods

### Animal Model of Brachial Root Avulsion

The animal experiments followed the role of the Chinese National Institutes of Health Guide for the Care and Use of Laboratory and Animal experiment and were approved by the Committee on the Use of Live Animals for Teaching and Research of the Zhongshan School of Medicine in Sun Yat-sen University. Totally, 38 of the adult male Sprague-Dawley rats (150–180 g) were used in the study. The procedure of the avulsion of the right C5-T1 ventral and dorsal roots was performed following the previous publications (Wu et al. 1994; Zhou and Wu 2006). After anesthetized with the intraperitoneal injections of 10 % chloral hydrate (350 mg/kg), the rats were operated in supine position under a surgical microscope. After identifying the right brachial plexus in the cervical region, the ventral and dorsal roots of C5, C6, C7, C8, and T1 were separated one by one. The extravertebral root avulsion was carried out by pulling out both dorsal and ventral roots of each spinal nerve with microhemostatic forceps. All of distal parts of the avulsed rootlets were taken out and examined under the microscope to ensure successful avulsion surgery. Then, the muscle, fascia, and skin were sutured successfully in layers and the animals were taken care with a high standard in order to minimize pain and discomfort. At the end of the first week (*n* = 12), the second week (*n* = 8), and the fourth week (*n* = 8) of the BPRA, all of the animals were kept alive.

### Retrograde Labeling of the Injured Spinal Motoneurons with Fluorogold

Following the procedures described in the previous publications (Tsujino et al. 2000; Fu et al. 2014), the fluorogold (FG) retrograde labeling of the avulsion-injured motoneurons was performed on five adult male SD rats at 3 days before the brachial plexus root avulsion surgery. Another five rats were used as the sham control. The rats were anesthetized with the intraperitoneal injections of 10 % chloral hydrate (350 mg/kg) and operated in supine position under a surgical microscope. After identifying the C5, C6, C7, C8, and T1 nerve roots of the right brachial plexus, the C7 and C8 spinal nerve roots were injected with 2 % FG (2 % *w*/*v*, Fluorochrome, Inc., Englewood, CO, USA). The FG solution (2.0 μl for 60 s) was slowly injected under the epineurium into the proximal stumps of the C7 and C8 nerves using a micropipette. Then, the injection site was clamped with microforceps for another 10 s to ensure all of the axons were cut. To insure the maximal labeling, the clamp site was loosening in order to let all of the axoplasm together with the FG solution to fill in and retrograde transported by the injured axons. The injection site was just proximal to the formation of the middle and inferior nerve stems of the right brachial plexus. Finally, the muscle, fascia, and skin were sutured successively in layers. Three days later, all of the rats in this study were anesthetized again. According to our previous study (Fu et al. 2014), all rats received a laminectomy of C6 and C7 vertebrae under the surgical microscope. The dura mater and the subarachnoid space were opened to expose the dorsal and ventral roots of the C7 and C8 spinal nerves. Then, all of the dorsal and ventral rootlets were pulled out using a micropipette hook. Muscles, fascia, and skin were then sutured successively in layers. The animals were allowed to recover until they awakened and were subsequently returned to their cages. For the sham control rats, all of the surgical procedures except the injection of FG and avulsion of C7 and C8 roots were also conducted as the FG-labeled rats.

### Grasping Tests

The grasping test was designed to test the strength of the finger flexion innervated by the median nerve (Bertelli and Mira 1995; Bertelli et al. 1995; Papalia et al. 2003) at 10 days and 4 weeks postinjury. When the tail of the rat was lifted, the paws would grasp the grid to get body balance. During grasping test, the tail of the rat was gently lift until only the tested forepaw firmly grasps the grip which was connected to an ordinary electronic balance. At the moment of the paw loosed its grip, the value in grams (g) in the electric balance was recorded. The highest value among five measurements, with 5-min time interval, was considered as the grasping strength. Both of the ipsilateral and contralateral forepaws of the BPRA-injured rats were tested. The grasping strength of the ipsilateral forepaws at 4 weeks was counted as 0 g since all of the BPRA-injured rats lost their ipsilateral forepaws.

### Tissue Preparation

All of the animals were killed by a lethal dose of chloral hydrate. Transcardial perfusion of normal saline followed by 4 % paraformaldehyde (PFA) in 0.1 M phosphate buffer (PB, pH 7.4) was carried out. Only the rats with complete avulsion of all of the dorsal and ventral rootlets of the C6, C7, and C8 spinal nerves were used in the following morphological studies. All of the C7 spinal segments, which were defined as the region between the uppermost rootlet of the C7 and the lowermost rootlet of the C7 of the contralateral spinal cord, were harvested and postfixed in 4 % PFA and followed by 30 % (*v*/*v*) sucrose in PB solution at 4 °C overnight. The serial coronal and transverse sections of the spinal cord (35 μm) were cut on a cryostat (Leica, Germany) and collected in the 0.01 M PBS. The serial transverse sections were collected in the 0.01 M PB in the dark to be prepared for ATF-3 immunofluorescence of the FG-labeled injured motoneurons. Every third transverse section of the spinal cord was used for FG-labeling investigation only, ATF-3 immunofluorescence, and neutral red stain (*n* = 5 at 24-h postinjury time point). The serial coronal sections of the spinal cord were used for ATF-3 and NeuN immunofluorescence double labeling (*n* = 4, for the first week time point). The serial transverse sections were also prepared, and every third section of the spinal cord was used for ATF-3 immunohistochemistry, ATF-3 and NeuN immunofluorescence double labeling, and NADPH-diaphorase (NADPH-d) histochemistry plus neutral red stain (*n* = 5 for normal control, *n* = 8 for 1-, 2-, and 4-week postinjury time points).

### ATF-3 and NeuN Immunofluorescence

The immunofluorescence procedures followed our previous studies (Wu 1996; Tang et al. 2014). First, sections were blocked with 0.3 % Triton X-100 (BBI, Canada) and 3 % bovine serum albumin (BSA, Jetway, China) in 0.1 M PBS at room temperature for 30 min. Second, sections were incubated with the primary antibodies (Millipore Cooperation, Billerica, MA, USA): the rabbit anti-ATF-3 antibody (1:500) for 72 h or the mouse anti-NeuN antibody (1:500) for 6 h at 4 °C in the dark. For double labeling of ATF-3 and NeuN, the anti-NeuN antibody was added to the anti-ATF-3 antibody solution at the final 6 h of the incubation. After washing in PBS for three times, the sections were incubated with the fluorescence secondary antibodies (Sigma, Saint Louis, MO, USA): the tetramethylrhodamine isothiocyanate (TRITC)-conjugated anti-rabbit IgG (1:400) or FITC-conjugated anti-mouse IgG (1:200) at room temperature for 2 h in the dark. The sections, after washing in PBS, were mounted on gelatin-coated glass slides, coverslipped in 0.1 M PBS containing 50 % glycerin, and examined via a fluorescence microscope. The images of the ipsilateral and contralateral C7 ventral horns were visualized under a fluorescence microscope and captured (×4, ×10 and ×20 lens) with a Lucida camera attached to the fluorescence microscope (Zeiss, Oberkochen, Germany). Control experiments included omission of the primary or secondary antibodies.

### NADPH-d Histochemistry Plus Neutral Red Counterstain

In adult rat model of BPRA, previous studies have proven that NADPH-d stains exactly the same population of affected motoneurons visualized by nNOS immunohistochemistry and nNOS in situ hybridization (Bredt et al. 1991; Wu 1993). Following the previous procedure, free-floating sections were incubated in 10 ml 0.1 M Tris-HCl (pH 8.0) containing 0.2 % Triton X-100, 10 mg NADPH (Sigma, Saint Louis, MO, USA),and 2.5 mg nitro-blue tetrazolium (Sigma, Saint Louis, MO, USA) at 37 °C for about 60 min and then washed with 0.1 M PB three times. The stained sections were mounted onto slides and counterstained with 1 % neutral red (NR, Sigma, Saint Louis, MO, USA). The images of the ipsilateral and contralateral C7 ventral horns were visualized under a microscope and captured (×10 and ×20 lens) with a Lucida camera attached to the microscope (Motic, China). These sections were used to count the numbers of nNOS-positive and surviving motoneurons.

### Motoneuron Counting and Statistics

Counting of the number of the motoneurons in the transverse sections of the C7 and C8 spinal segments was performed under a ×20 objective lens by two people who were blind to rats’ subgroups as in the previous studies (Wu 1993; Wu and Li 1993; Cheng et al. 2013). The FG and ATF-3 double-labeled motoneurons were counted, the percentage of colocalization of either FG with ATF-3, or ATF-3 with FG in ipsilateral ventral horn motoneurons were calculated as the previous publication (Tsujino et al. 2000). The number of the survived motoneurons was counted with only those neurons with both the nucleolus in the nucleus and Nissl bodies in the cytoplasm stained with neutral red (NR). The total number of the survival motoneurons on the contralateral side was set at 100 % and served as an internal control for each section. The number of the nNOS-positive motoneurons was counted with only the motoneurons showing a cytoplasm stained with NADPH-d with visible nuclei. The number of nNOS-positive motoneurons in the ipsilateral ventral horn was expressed as a percentage of the survival motoneurons in the contralateral ventral horn in the same section. The number of ATF-3-positive and the NeuN-positive motoneurons were separately counted in both ipsilateral and contralateral ventral horns. Since there were no ATF-3 positive in the contralateral and almost none NeuN-positive reactions in the ipsilateral ventral horns, the number of ATF-3-positive motoneurons in the ipsilateral ventral horn was expressed as a percentage of the NeuN-positive motoneurons in the contralateral ventral horn in the same section (Zhou et al. 2008; Wang et al. 2011). The average values (*X*) and standard errors of the mean deviations (SE) were presented as mean ± SEM in the present study. The statistical calculation and data handling were performed by one-way ANOVA followed by the post hoc Bonferroni test using SPSS software (version 16.0; SPSS, Chicago, IL, USA). Significance was set at *p* < 0.05.

## Results

### Avulsion-Induced Motor Functional Loss of the Affected Forepaws

**Fig. 1:**
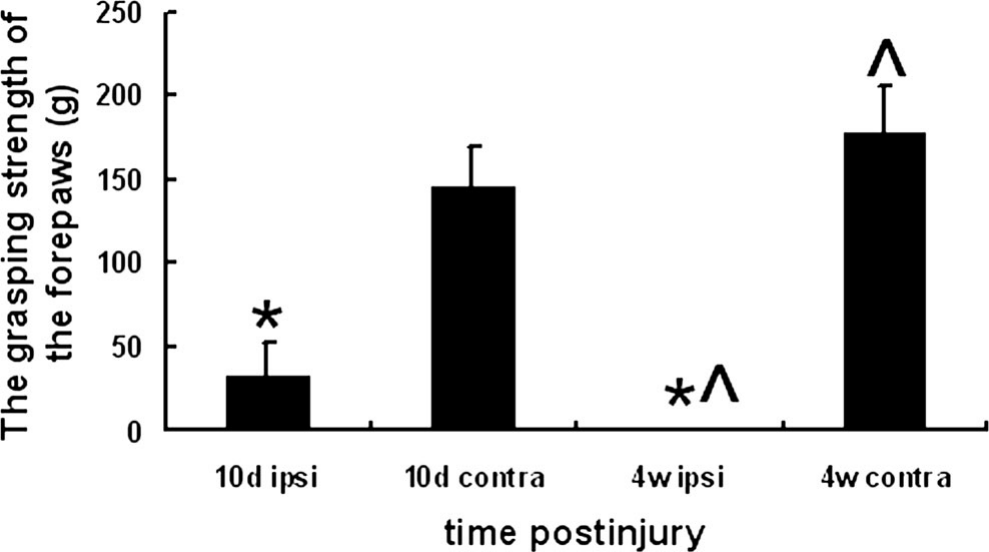
The grasping test of the forepaws of the rats after brachial roots avulsion. The average grasping strength (*g*) of the ipsilateral and contralateral forepaws in BPRA-injured rats at 10 days and 4 weeks after right brachial roots avulsion. *p < 0.05 compared to that of the contralateral forepaws at the same time point, ^*p* < 0.05 compared to that at 10 days in the same side of the upper limbs

In order to confirm the success of the unilateral BPRA of the experimental animals, the grasping test was used to compare the motor behavior of the hands of the affected rats. The results showed that the rats that suffered from the BPRA did not recovery active finger flexion till the end of the study. Quantitative comparison which showed the strength of finger flexion was significantly lower in ipsilateral than that of the contralateral forepaw on the 10th day postinjury (Fig. 1). At the end of the 4 weeks postinjury, all of the BPRA-injured rats totally lost the strength of finger flexion (0 g) ipsilaterally because their ipsilateral forepaws had been bit out. However, the strength of finger flexion of the contralateral forepaws was significantly increased in rats at 4 weeks when compared to those at 10 days postinjury (Fig. 1). This result confirmed the successful unilateral BPRA of the experimental rats in the following studies.

### Avulsion Induced an Immediate Expression of ATF-3 in the Nuclei of the Fluorogold Retrograde Labeled Injured Motoneurons Following C7 and C8 Root Avulsion

**Fig. 2:**
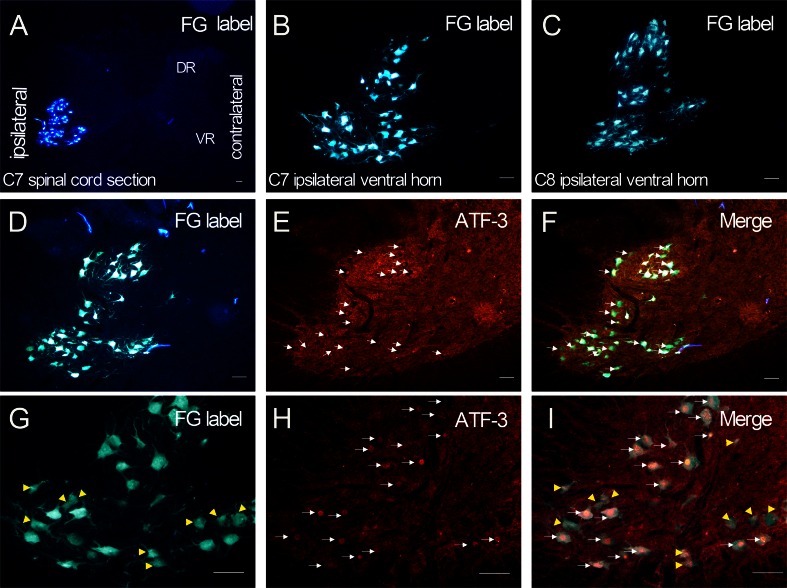
Double labeling for fluorogold and ATF-3 in the injured motoneurons in the ventral horns at 24 h following the right C7 and C8 roots avulsion. The fluorogold retrograde labeling was carried out 3 days before C7 and C8 roots avulsion; the C7–C8 spinal cords were taken out at 24 h following avulsion. Only the ipsilateral avulsion-injured ventral horn motoneurons, not other cells, were labeled by fluorogold injected into the C7 and C8 nerve roots (**a**). Moreover, almost all of the motoneurons in the ipsilateral C7 (**b**) and C8 (**c**) spinal segments were traced by the fluorogold. In the double labeling of fluorogold and ATF-3 sections of the C7–C8 spinal segments, a robust ATF-3 expression was detected in the nuclei of the ipsilateral ventral horn motoneurons (*white arrow*, **e**, **h**). Most of the fluorogold-labeled motoneurons (**d**, **g**) were labeled by ATF-3 in the nuclei; and all of the ATF-3 positive motoneurons were labeled by the fluorogold (**f**, **i**). Some of the fluorogold-labeled motoneurons were not labeled with ATF-3 (yellow arrow, g, i). *Bar* = 100 μm

At the end of the 3 days of the FG retrograde labeling, the C7 and C8 spinal sections were examined under the fluorescent microscope. The FG-labeled motoneurons indicate that the retrograde FG tracer was taken up by the cut end of the axons in the C7 and C8 nerve roots and transported back to the cytoplasm of the motoneurons’ bodies in the C7 and C8 ventral horns (Fig. 2), so that the FG-labeled motoneurons represented the injured motoneurons.

Our result showed that only the injured motoneurons in ipsilateral ventral horn were labeled by FG (Fig. 2a). Moreover, almost all of the injured motoneurons, including their cell body and the processes, were labeled by the FG in the ipsilateral C7 (Fig. 2b) and C8 (Fig. 2c) ventral horns. At 24 h following C7 and C8 root avulsion, a robust ATF-3 expression was detected in the nuclei of the ipsilateral ventral horn motoneurons (white arrows in Fig. 2e, h). Those FG-labeled motoneurons without the clear nuclei were ATF-3 negative (yellow arrows in Fig. 2g, i). Quantitative analysis showed that 100 % of the ATF-3 positive motoneurons were FG-labeled (Fig. 2f, i), and about 60 % (58.9 % ± 4.71 %) of the FG-labeled motoneurons were ATF-3 positive (white arrows in Fig. 2f, i).

### Avulsion Induced a Sustained Expression of ATF-3 in the Nuclei but Almost Disappearance of the NeuN Expressions in the Injured Motoneurons Within 4 weeks of BPRA

**Fig. 3:**
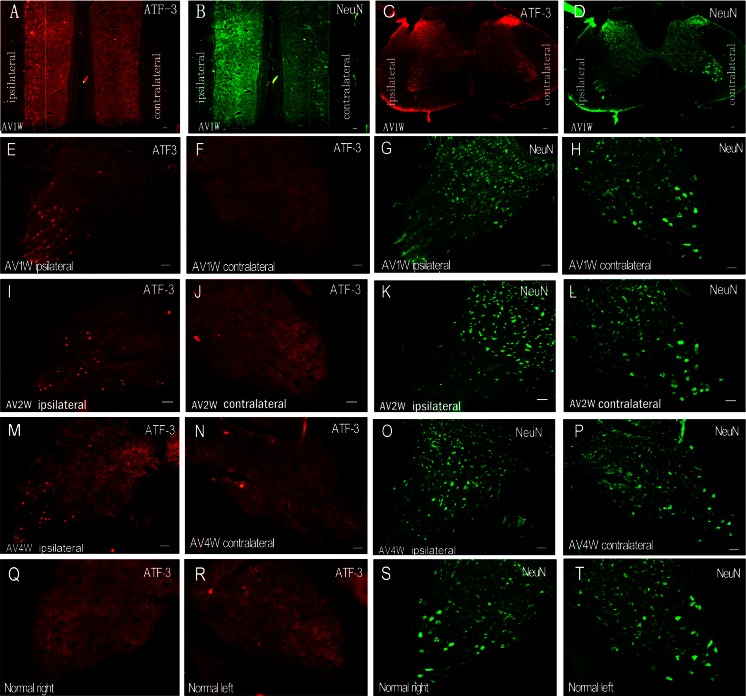
Immunofluorescence double labeling of ATF-3 (*red*) and NeuN (*green*) in the injured motoneurons within 4 weeks following right BPRA. **a**–**h** At the first week of BPRA, the avulsion-induced absence of NeuN and induction of ATF3 occurred only in the ipsilateral ventral horns which showed in both the coronal (**a**, **b**) and transverse (**c**, **d**) sections of the C7 spinal segments. At the end of the first (**e**–**h**), the second (**i**–**l**), and the fourth (**m**–**p**) weeks of BPRA, avulsion resulted in a remarkable ATF-3 induction with the positive particles located in the nuclei of the injured motoneurons in the ipsilateral (**e**, **i**, **m**) but not in the contralateral (**f**, **j**, **n**) ventral horns; in contrast, the NeuN positive immunoreactions only did not occurred in the motoneurons but could be detected in other neuronal populations in the ipsilateral ventral horns (**g**, **k**, **o**). The NeuN-positive particles could be found in the motoneurons in the contralateral ventral horns (**h**, **l**, **p**). There were no ATF-3 positive reactions in the spinal cord (**q**, **r**), but all of the neurons in bilateral ventral horns were NeuN positive (**s**–**t**) in the normal control rat. *Bar* = 100 μm

The positive immunoreactive ATF-3 particles continuously existed and could be checked out (red, Fig. 3) in the nuclei of the ipsilateral injured motoneurons on the C7–C8 ipsilateral ventral horns at the end of the first, (Fig. 3a, c, e), the second (Fig. 3i), and the fourth (Fig. 3m) week of the BPRA. In the contralateral ventral horns of the same section of the spinal cord (Fig. 3a, c, f, j, n), as well as that in normal control rats (Fig. 3q, r), there is no ATF-3 expression in the nuclei of the ventral horn motoneurons.

For the NeuN imunofluororescence reaction, the results showed that the positive immunoreactions of NeuN antibody exhibited concentrated staining in the nuclei and diffuse cytoplasm staining in both processes and the cell bodies of all of the spinal neurons in the contralateral ventral horns of the injured spinal segments (green, Fig. 3b, d, h, l, p), just like the NeuN expression pattern in the normal control rat (Fig. 3s, t). But the NeuN expression could not be detected in the injured motoneurons in Rexed layer IX of the ipsilateral ventral horns at the end of the first (Fig. 3b, d, g), the second (Fig. 3k), and the fourth (Fig. 3o) week after BPRA.

Since the NeuN expression disappeared in the ipsilateral ventral horn motoneurons, the number of the NeuN-positive neurons in the contralateral ventral horn was used as the internal control to assess the intensity of the ATF-3 immunofluorescence reactions in the NeuN and ATF-3 double-labeling sections. The result showed that the induced ATF-3-positive motoneurons in the ipsilateral ventral horn were 62.1 ± 6.14 % of the NeuN-positive motoneurons in the contralateral ventral horn in the same C7 sections at the end of the second week of BPRA.

### Avulsion-Induced Death of the Spinal Motoneurons

**Fig. 4:**
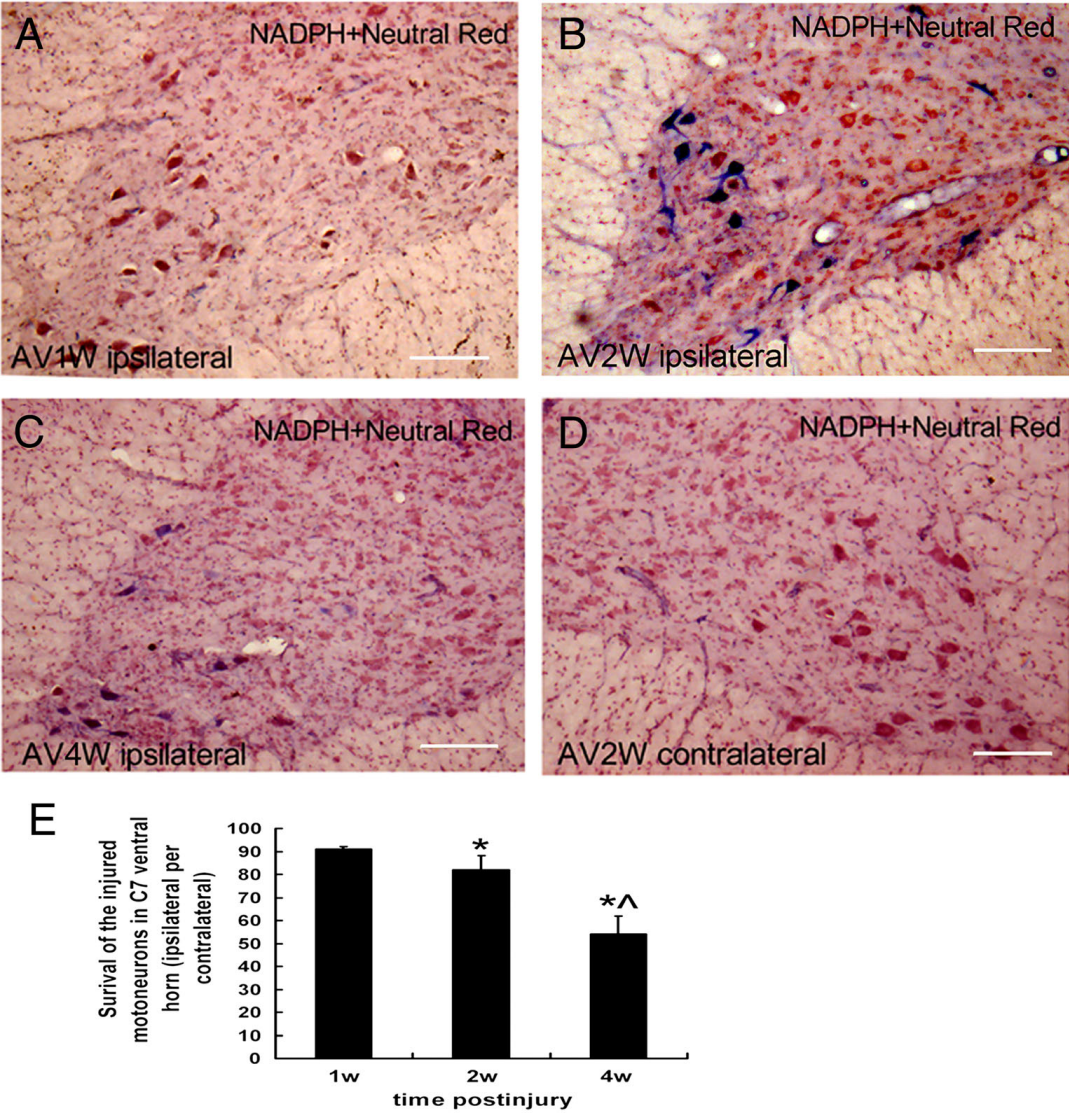
NADPH-d (*blue*) plus neutral red (*red*) counterstaining in the injured motoneurons, within 4 weeks following right BPRA. Avulsion-induced nNOS expressions were obvious, shown in the ipsilateral injured motoneurons from the second week (**b**) to the fourth (**c**) week but did not occurred at the first week (**a**) of BPRA. The loss of the injured motoneurons was not obvious within the first 2 weeks but occurred at the fourth week (**c**) in the ipsilateral ventral horn (**d**). The avulsion did not cause the nNOS expression in the contralateral ventral horn motoneurons. *Bar* = 100 μm (**e**). Quantitative analysis showed the BPRA resulted in the continuous reduction of the survival rate of the injured motoneurons within 4 weeks of BPRA. *p < 0.05 compared to that at the first week; ^*p* < 0.05 compared to that at the second week

In the present study, the neutral red-stained neurons with typical Nissl bodies in Rexed’s lamina IX were counted and considered as the survival motoneurons in both the contralateral and ipsilateral C7 ventral horns (Fig. 4). The number of motoneurons in the contralateral Rexed’s lamina IX was not affected by the BPRA since our previous study had demonstrated that the number of survival motoneurons in the contralateral C7 ventral horns stained by the neutral red was not significantly different among the avulsion-injured rats (226.4 ± 5.81), sham-operated control (227.4 ± 2.79), and normal (227.8 ± 3.19) adult SD rats (Zhao et al. 2012). Therefore, it was used as an internal control for each section in the present study. In BPRA rats, the survival motoneurons in the ipsilateral Rexed’s lamina IX were 91.04 ± 1.05 % of that of the contralateral at the first week (Fig. 4a), further decreased to 82.06 ± 6.05 % of that of the contralateral at the second week (Fig. 4b). At the end of the fourth week of BPRA, there was only 54.06 ± 7.98 % of the motoneurons in the ipsilateral (Fig. 4c) compared to 100 % of the motoneurons in the contralateral (Fig. 4d) Rexed’s lamina IX survived the injury. The survival rate was significantly different between those at any two time points (all *p* < 0.05, Fig. 4e). The result showed that the BPRA induced death of the affected motoneurons.

### Avulsion-Induced a Delayed nNOS Expressions in the Affected Motoneurons

Since, the oxidative stress has been considered to contribute to the death of the avulsion-injured motoneuron death and the nNOS was mostly used as a marker for the oxidative stress inside spinal motoneurons (Martin, et al. 2005; Wu and Li 1993; Yu 2002) in the filed of the avulsion injury. We also investigated the nNOS expressions in the ipsilateral ventral horns (Fig. 4). Here, the NADPH-d stained (blue in spinal cord sections, Fig. 4b, c) was represented as the nNOS-positive motoneurons. Root avulsion did not induce nNOS expression in the affected motoneurons within the first week of BPRA (Fig. 4a). At the second (Fig. 4b) and the fourth (Fig. 4c) week of BPRA, the nNOS was obviously induced to express in the cytoplasm and the processes of the injured motoneurons. There were no nNOS-positive reactions that occurred in contralateral ventral horn motoneurons (Fig. 4d). Quantification of the number of the nNOS-positive motoneurons showed that there were 28.11 ± 10.10 % motoneurons expressed nNOS protein at the end of the second week of BPRA.

## Discussion

The grasping test, the most commonly used test for motor behavior, was designed to quantify the strength of the finger flexion (Bertelli and Mira 1995; Bertelli et al. 1995) which is controlled by the median nerve (Papalia et al. 2003), a branch formed by the C7 and C8 roots of the brachial plexus in the rats. In the present study, all of the ventral and dorsal roots of both C7 and C8 spinal segments were avulsed and removed. Thus, the reduced grasping strength of the ipsilateral forepaws at 10 days postlesion confirmed the successful surgery of the unilateral BPRA. At the fourth week of the BPRA, almost all of the ipsilateral forepaws were bitten by the injured rats themselves because of the pain. This result was coincident with the previous studies of the same animal models of BPRA (Gu et al. 2004; Fu et al. 2014). Furthermore, the present result of avulsion-induced loss of the ipsilateral motoneurons in the Rexed’s lamina IX in C7 segments supported the recent studies which have demonstrated the lateral motoneuron column in the cervical spinal cord and contributes to the grasping behavior (Stepien et al. 2010; Bui et al. 2013).

Previous study has suggested that neither ChAT nor NeuN could be a single specific phenotypic marker to assess the fate of injured motoneurons in axonal injury (McPhail et al. 2004a) since the facial nerve resection in the rat resulted in an absence of NeuN immunoreactivity in facial motoneurons until 28 days postinjury (McPhail et al. 2004a). Our present data of NeuN immunofluorescence reaction of the injured spinal cord supported the previous studies. Our results showed the absence of the NeuN immunoreactivity that occurred in BPRA-injured cervical spinal motoneurons within the first 4 weeks postinjury in the rat. Furthermore, our data presented the BPRA-induced disappearance of NeuN that only occurred in ipsilateral spinal motoneurons. We contributed this result to the avulsion-induced disturbance of the biochemical process in the cell bodies of the motoneurons. Because avulsion results in the disconnection of the axons of the spinal motoneurons from their targeted skeletal muscles, which caused the deprivation of the trophic factors released both by Schwann cells of the distal ends of the injured axons and the targeted skeletal muscles (Fernandes and Tetzlaff 2000). Deprivation of the target-derived neurotrophic factors resulted in several interrelated damage processes in motoneurons including morphological alterations, biochemical disturbances, gene expression dysregulation, metabolic changes, and cell death (Li et al. 1995; Eggers et al. 2010; Fernandes and Tetzlaff 2000; Gu et al. 2004; Wu 1996). NeuN is a transcription factor, participating in the biochemical process of the proteins, which is expressed in the nucleus and cytoplasm in the healthy mature neurons (Mullen et al. 1992). Under the pathological conditions that are fatal to the neurons such as in BPRA, cerebral ischemia, and hypoxia and trauma, the NeuN immunoreactivity has been reported to decrease in the injured neurons (Igarashi et al. 2001; Davoli et al. 2002; Cheng et al. 2014; Cui et al. 2014). The injury-induced loss of NeuN immunoreactivity has been considered to be due to the depletion of NeuN protein or loss of its antigenicity (McPhail et al. 2004a, b; Unal-Cevik et al. 2004).

The present data suggest that the ATF-3 could be a specific phenotypic marker of BPRA-injured motoneurons since BPRA resulted in a sustained expression of ATF-3 in the injured spinal motoneurons. Our data of the fluorogold and ATF-3 double labeling conformed that the avulsion-induced ATF-3 expression occurred only in the injured motoneurons, not in other cells, because all of the ATF-3 positive motoneurons were FG positive. In the present study, the FG-labeled motoneurons represented the injured motoneurons. Because the FG-labeled motoneurons indicate that the retrograde FG tracer had been taken up by the cut end of the axons in the C7 and C8 nerve roots and transported back to the cytoplasm of the motoneurons’ bodies in the C7 and C8 ventral horns. However, our present data showed that 60 %, not 100 %, of the FG-positive motoneurons were ATF-3 positive. We contribute this difference to the specific localization of the ATF-3 immunoreactivity particles. The ATF-3 expression occurred only in the nuclei of the neurons, and not all of the nuclei of the motoneurons could be presented in the same section of the spinal cord. A previous study had showed ATF-3-positive immunoreaction remained in the surviving and regenerating motoneurons in avulsion injury; thus, they suggested ATF-3 as a regenerative marker of the affected motoneurons (Linda et al. 2011). But in our present study, the distal part of the axons of the affected motoneurons was totally removed; the affected motoneurons underwent the process of degeneration but not regeneration (Fu et al. 2014). Thus, we suggested that ATF-3 marks the injured and degenerating spinal motoneurons.

As a member of the activating transcription factor/cAMP responsive element-binding protein (ATF/CREB) family of transcription factors, ATF-3 binds to the specific DNA and forms homodimers or heterodimers with other bZIP-containing proteins via the leucine zipper region; thus, ATF-3 may act as either a repressor or an activator in the regulation of transcription (Thompson et al. 2009). ATF-3 was thought to function as an early responder to link various downstream events of the signaling pathways involved in the alteration of multi-cellular behavior (Hai et al. 2010). Previous studies have demonstrated that the significance of ATF-3 upregulation in the immediate response to axonal injury depends on its downstream target genes (Sheng and Greenberg 1990; Linda et al. 2011). In the present study, both ATF-3 and nNOS induced to express in the injured motoneurons, however, the nNOS expression occurred about 2 weeks later than that of the ATF-3. The correlation of the expressions between ATF-3 and nNOS in the present study was similar to that between the phospho-c-jun and nNOS in the previous studies (Wang et al. 2011; Li et al. 2015). The phosphorylation of the c-jun in the BPRA-injured motoneurons was demonstrated to contribute to the death of the injured motoneurons and also thought to regulate the downstream nNOS gene expression in BPRA-injured motoneurons (Yuan et al. 2010; Cheng et al. 2013, 2014). Furthermore, previous study has found that a function of ATF-3 in neurons under death stress, just like the spinal motoneurons under root avulsion in the present study, is to inhibit the mitogen-activated kinase kinase kinase 1 (MEKK1)—c-jun N-terminal kinase (JNK)-induced apoptosis (Nakagomi et al. 2003; Greer et al. 2011). Therefore, we suggest that nNOS might be one of the indirect downstream target genes of the ATF-3 in BPRA injury.

The nNOS-positive motoneuron quantification in the present study was similar to those of the previous studies at the same time point by using the same animal model (Wu 1993; Zhao et al. 2012). It indicates that the present animal model was believable. The previous studies have demonstrated the time pattern of the de novo nNOS expression in BPRA-injured motoneurons which started from 10 days, peaked at 3 weeks, then reduced until the end of study, and all of nNOS-positive motoneurons are the survived injured motoneurons (Wu 1993). The function of BPRA-induced upregulation of nNOS in the affected motoneurons have been suggested to depend on the overproduction of nitric oxide (NO) and the oxidative stress to affected motoneurons (Martin et al. 1999; Yu 2002; Martin et al. 2005). However, other studies demonstrated the upregulation of nNOS gene is beneficial for the injured motoneuron survival as early as within the first 2 weeks (Zhou and Wu 2006; Wang et al. 2011). For the mechanism of nNOS gene in the BPRA injury, it needs more studies to explore later on.

In conclusion, the present data suggest that ATF-3, but not NeuN, is a stable molecular marker to mark the injured spinal motoneurons in axonal injury.

## Abbreviations

ChAT: Choline acetyltransferase
NeuN: Neuronal nuclei DNA-binding protein
ATF-3: Activating transcriptional factor
nNOS: Neuronal nitric oxide synthase
FG: Fluorogold
PFA: Paraformaldehyde
BSA: Bovine serum albumin
TRITC: Tetramethylrhodamine isothiocyanate
ANOVA: One-way analysis of variance
NO: Nitric oxide

## References

[CR1] Bertelli JA, Mira JC (1995). The grasping test: a simple behavioral method for objective quantitative assessment of peripheral nerve regeneration in the rat. J Neurosci Methods.

[CR2] Bertelli JA, Taleb M, Saadi A, Mira JC, Pecot-Dechavassine M (1995). The rat brachial plexus and its terminal branches: an experimental model for the study of peripheral nerve regeneration. Microsurgery.

[CR3] Bredt DS, Glatt CE, Hwang PM, Fotuhi M, Dawson TM, Snyder SH (1991). Nitric oxide synthase protein and mRNA are discretely localized in neuronal populations of the mammalian CNS together with NADPH diaphorase. Neuron.

[CR4] Brown GC (2010). Nitric oxide and neuronal death. Nitric Oxide.

[CR5] Bui TV, Akay T, Loubani O, Hnasko TS, Jessell TM, Brownstone RM (2013). Circuits for grasping: spinal dI3 interneurons mediate cutaneous control of motor behavior. Neuron.

[CR6] Cheng X, Fu R, Gao M, Liu S, Li YQ, Song FH, Bruce IC, Zhou LH, Wu W (2013). Intrathecal application of short interfering RNA knocks down c-jun expression and augments spinal motoneuron death after root avulsion in adult rats. Neuroscience.

[CR7] Cheng X, Luo H, Hou Z, Huang Y, Sun J, Zhou L (2014). Neuronal nitric oxide synthase, as a downstream signaling molecule of c-jun, regulates the survival of differentiated PC12 cells. Mol Med Rep.

[CR8] Cui Y, Masaki K, Yamasaki R, Imamura S, Suzuki SO, Hayashi S, Sato S, Nagara Y, Kawamura MF, Kira J (2014). Extensive dysregulations of oligodendrocytic and astrocytic connexins are associated with disease progression in an amyotrophic lateral sclerosis mouse model. J Neuroinflammation.

[CR9] Dasen JS, Tice BC, Brenner-Morton S, Jessell TM (2005). A Hox regulatory network establishes motor neuron pool identity and target-muscle connectivity. Cell.

[CR10] Davoli MA, Fortunis J, Tam J, Xanthoudakis S, Nicholdon D, Ng GS, Ng GYK, Xu D (2002). Immunohistochemical and biochemical assessment of caspase-3 activation and DNA fragmentation following transient focal ischemia in the rat. Neuroscience.

[CR11] Eggers R, Tannemaat MR, Ehlert EM, Verhaagen J (2010). A spatio-temporal analysis of motoneuron survival, axonal regeneration and neurotrophic factor expression after lumbar ventral root avulsion and implantation. Exp Neurol.

[CR12] Fernandes KJ, Tetzlaff W (2000) Gene expression in axotomized neurons: identifying the intrinsic determinants of axonal growth. In: Ingoglia NA, Murray M (eds) Axonal Regeneration in the Central Nervous System, New York Marcel Dekker, pp 219–266

[CR13] Forstermann U, Sessa WC (2012). Nitric oxide synthases: regulation and function. Eur Heart J.

[CR14] Friese A, Kaltschmidt JA, Ladle DR, Sigrist M, Jessell TM, Arber S (2009). Gamma and alpha motor neurons distinguished by expression of transcription factor Err3. Proc Natl Acad Sci U S A.

[CR15] Fu R, Tang Y, Ling ZM, Li YQ, Cheng X, Song FH, Zhou LH, Wu W (2014). Lithium enhances survival and regrowth of spinal motoneurons after ventral root avulsion. BMC Neurosci.

[CR16] Goetz C, Pivetta C, Arber S (2015). Distinct limb and trunk premotor circuits establish laterality in the spinal cord. Neuron.

[CR17] Greer JE, McGinn MJ, Povlishock JT (2011). Diffuse traumatic axonal injury in the mouse induces atrophy, c-Jun activation, and axonal outgrowth in the axotomized neuronal population. J Neurosci.

[CR18] Gu HY, Chai H, Zhang JY, Yao ZB, Zhou LH, Wong WM, Bruce I, Wu WT (2004). Survival, regeneration and functional recovery of motoneurons in adult rats by reimplantation of ventral root following spinal root avulsion. Eur J Neurosci.

[CR19] Hai T, Wolford CC, Chang YS (2010). ATF3, a hub of the cellular adaptive-response network, in the pathogenesis of diseases: is modulation of inflammation a unifying component?. Gene Expr.

[CR20] Hoang TX, Nieto JH, Tillakaratne NJ, Havton LA (2003). Autonomic and motor neuron death is progressive and parallel in a lumbosacral ventral root avulsion model of cauda equina injury. J Comp Neurol.

[CR21] Igarashi T, Huang TT, Noble LJ (2001). Regional vulnerability after traumatic brain injury: gender differences in mice that over express human copper, zinc superoxide dismutase. Exp Neurol.

[CR22] Kwon BK, Liu J, Oschipok L, Tetzlaff W (2002). Reaxotomy of chronically injured rubrospinal neurons results in only modest cell loss. Exp Neurol.

[CR23] Kwon BK, Liu J, Messerer C, Kobayashi NR, McGraw J, Oschipok L, Tetzlaff W (2002). Survival and regeneration of rubrospinal neurons 1 year after spinal cord injury. Proc Natl Acad Sci U S A.

[CR24] Li L, Wu W, Lin LF, Lei M, Oppenheim RW, Houenou LJ (1995). Rescue of adult mouse motoneurons from injury-induced cell death by glial cell line-derived neurotrophic factor. Proc Natl Acad Sci U S A.

[CR25] Li K, Cao RJ, Zhu XJ, Liu XY, Li LY, Cui SS (2015) Erythropoietin attenuates the poptosis of adult neurons after brachial plexus root avulsion by downregulating JNK phosphorylation and c-Jun expression and inhibiting c-PARP cleavage. J Mol Neurosci 10.1007/s12031-015-0543-4 25877688

[CR26] Linda H, Skold MK, Ochsmann T (2011). Activating transcription factor 3, a useful marker for regenerative response after nerve root injury. Front Neurol.

[CR27] Martin LJ, Kaiser A, Price AC (1999). Motor neuron degeneration after sciatic nerve avulsion in adult rat evolves with oxidative stress and is apoptosis. J Neurobiol.

[CR28] Martin LJ, Chen K, Liu Z (2005). Adult motor neuron apoptosis is mediated by nitric oxide and Fas death receptor linked by DNA damage and p53 activation. J Neurosci.

[CR29] McPhail LT, McBride CB, McGraw J, Steeves JD, Tetzlaff W (2004). Axotomy abolishes NeuN expression in facial but not rubrospinal neurons. Exp Neurol.

[CR30] Mullen RJ, Buck CR, Smith AM (1992). NeuN, a neuronal specific nuclear protein in vertebrates. Development.

[CR31] Nakagomi S, Suzuki Y, Namikawa K, Kiryu-Seo S, Kiyama H (2003). Expression of the activating transcription factor 3 prevents c-Jun N-terminal kinase-induced neuronal death by promoting heat shock protein 27 expression and Akt activation. J Neurosci Off J Soc Neurosci.

[CR32] Novikov L, Novikova L, Kellerth JO (1995). Brain-derived neurotrophic factor promotes survival and blocks nitric oxide synthase expression in adult rat spinal motoneurons after ventral root avulsion. Neurosci Lett.

[CR33] Oppenheim RW, Houenou LJ, Johnson JE, Lin LF, Li L, Lo AC, Newsome AL, Prevette DM, Wang S (1995). Developing motor neurons rescued from programmed and axotomy-induced cell death by GDNF. Nature.

[CR34] Papalia I, Tos P, Stagno d'Alcontres F, Battiston B, Geuna S (2003). On the use of the grasping test in the rat median nerve model: a re-appraisal of its efficacy for quantitative assessment of motor function recovery. J Neurosci Methods.

[CR35] Peddie CJ, Keast JR (2011). Pelvic nerve injury causes a rapid decrease in expression of choline acetyltransferase and upregulation of c-Jun and ATF-3 in a distinct population of sacral preganglionic neurons. Front Neurosci.

[CR36] Rende M, Giambanco I, Buratta M, Tonali P (1995). Axotomy induces a different modulation of both low-affinity nerve growth factor receptor and choline acetyltransferase between adult rat spinal and brainstem motoneurons. J Comp Neurol.

[CR37] Sheng M, Greenberg ME (1990). The regulation and function of c-fos and other immediate early genes in the nervous system. Neuron.

[CR38] Stepien AE, Tripodi M, Arber S (2010). Monosynaptic rabies virus reveals premotor network organization and synaptic specificity of cholinergic partition cells. Neuron.

[CR39] Tang Y, Ling ZM, Fu R, Li YQ, Cheng X, Song FH, Luo HX, Zhou LH (2014). Time-specific microRNA changes during spinal motoneuron degeneration in adult rats following unilateral brachial plexus root avulsion: ipsilateral vs. contralateral changes. BMC Neurosci.

[CR40] Thompson MR, Xu D, Williams BR (2009). ATF3 transcription factor and its emerging roles in immunity and cancer. J Mol Med.

[CR41] Tsujino H, Kondo E, Fukuoka T, Dai Y, Tokunaga A, Miki K, Yonenobu K, Ochi T, Noguchi K (2000). Activating transcription factor 3 (ATF3) induction by axotomy in sensory and motoneurons: a novel neuronal marker of nerve injury. Mol Cell Neurosci.

[CR42] Unal-Cevik I, Kilinç M, Gürsoy-Ozdemir Y, Gurer G, Dalkara T (2004). Loss of NeuN immunoreactivity after cerebral ischemia does not indicate neuronal cell loss: a cautionary note. Brain Res.

[CR43] Wang LL, Zhao XC, Yan LF, Wang YQ, Cheng X, Fu R, Zhou LH (2011). C-jun phosphorylation contributes to down regulation of neuronal nitric oxide synthase protein and motoneurons death in injured spinal cords following root-avulsion of the brachial plexus. Neuroscience.

[CR44] Wu W (1993). Expression of nitric-oxide synthase (NOS) in injured CNS neurons as shown by NADPH diaphorase histochemistry. Exp Neurol.

[CR45] Wu W (1996). Potential roles of gene expression change in adult rat spinal motoneurons following axonal injury: a comparison among c-jun, off-affinity nerve growth factor receptor (LNGFR), and nitric oxide synthase (NOS). Exp Neurol.

[CR46] Wu W (2000) Chapter IX Response of nitric oxide synthase to neuronal injury. In: HWM Steinbusch JDV, Vincent SR (eds) Handbook of chemical neuroanatomy. Elsevier, pp 315–353

[CR47] Wu W, Li L (1993). Inhibition of nitric oxide synthase reduces motoneuron death due to spinal root avulsion. Neurosci Lett.

[CR48] Wu W, Li Y, Schinco FP (1994). Expression of c-jun and neuronal nitric oxide synthase in rat spinal motoneurons following axonal injury. Neurosci Lett.

[CR49] Yu WH (2002). Spatial and temporal correlation of nitric oxide synthase expression with CuZn-superoxide dismutase reduction in motor neurons following axotomy. Ann N Y Acad Sci.

[CR50] Yuan Q, Hu B, Wu Y, Chu TH, Su H, Zhang W, So KF, Lin Z, Wu W (2010). Induction of c-Jun phosphorylation in spinal motoneurons in neonatal and adult rats following axonal injury. Brain Res.

[CR51] Zhao XC, Wang LL, Wang YQ, Song FH, Li YQ, Fu R, Zheng WH, Wu W, Zhou LH (2012). Activation of phospholipase-Cγ and protein kinase C signal pathways helps the survival of spinal motoneurons injured by root avulsion. J Neurochem.

[CR52] Zhou L, Wu W (2006). Antisense oligos to neuronal nitric oxide synthase aggravate motoneuron death induced by spinal root avulsion in adult rat. Exp Neurol.

[CR53] Zhou LH, Han S, Xie YY, Wang LL, Yao ZB (2008). Differences in c-jun and nNOS expression levels in motoneurons following different kinds of axonal injury in adult rats. Brain Cell Biol.

